# Microwave Absorption of α-Fe_2_O_3_@diatomite Composites

**DOI:** 10.3390/ijms23169362

**Published:** 2022-08-19

**Authors:** Chenzhi Zhang, Dashuang Wang, Lichao Dong, Kailin Li, Yifan Zhang, Pingan Yang, Shuang Yi, Xingjian Dai, Changqing Yin, Zhilan Du, Xinfang Zhang, Quan Zhou, Zhiyu Yi, Jinsong Rao, Yuxin Zhang

**Affiliations:** 1 College of Material Science and Engineering, Chongqing University, Chongqing 400044, China; 2 Aerospace Institute of Advanced Materials & Processing Technology, Beijing 100074, China; 3 School of Automation, Chongqing University of Posts and Telecommunications, Chongqing 400065, China

**Keywords:** diatomite, α-Fe_2_O_3_, microwave absorption

## Abstract

A neoteric round sieve diatomite (De) decorated with sea-urchin-like alpha-type iron trioxide (α-Fe_2_O_3_) synthetics was prepared by the hydrothermal method and further calcination. The results of the electromagnetic (EM) parameters of α-Fe_2_O_3_-decorated De (α-Fe_2_O_3_@D) showed that the minimum reflection loss (RL_min_) of α-Fe_2_O_3_@D could reach −54.2 dB at 11.52 GHz and the matched absorber thickness was 3 mm. The frequency bandwidth corresponding to the microwave RL value below −20 dB was up to 8.24 GHz (9.76–18 GHz). This indicates that α-Fe_2_O_3_@D composite can be a lightweight and stable material; because of the low density of De (1.9–2.3 g/cm^3^), the density of α-Fe_2_O_3_@D composite material is lower than that of α-Fe_2_O_3_ (5.18 g/cm^3^). We found that the combination of the magnetic loss of sea-urchin-like α-Fe_2_O_3_ and the dielectric loss of De has the most dominant role in electromagnetic wave absorption and loss. We focused on comparing the absorbing properties before and after the formation of sea-urchin-like α-Fe_2_O_3_ and explain in detail the effects of the structure and crystal shape of this novel composite on the absorbing properties.

## 1. Introduction

At present, with the widespread application and rapid development of radio equipment, electromagnetic (EM) interference, electromagnetic radiation and electromagnetic compatibility have become an important issue. On the one hand, they endanger the health of human and electronic equipment, On the other hand, they affect the development of the modern military [1,2,3,4,5,6]. Electromagnetic wave-absorbing materials (EWAM) have been extensively used in many areas of social life, for example in reducing electromagnetic radiation. In the future, the EWAM should have the characteristics of excellent electromagnetic absorption performance, thin thickness, a wide, effective absorbing bandwidth (EAB) and being of light weight to satisfy different application requirements. It is well known that the combination of different compounds which have excellent microwave properties has led to new composite materials which have earned great technological interest in recent years. The addition of a second phase can significantly improve the electronic properties of the resulting composite materials: Co_0.5_Ni_0.5_Ga_0.01_Gd_0.01_Fe_1.98_O_4_/ZnFe_2_O_4_ spinel ferrite nanocomposites [7] and carbon nanotubes/BaFe_12−x_Ga_x_O_19_/epoxy composites [8]. There are various types of ferrites based on their crystal structure, including Fe_3_O_4_ [9], gamma-Fe_2_O_3_ (γ-Fe_2_O_3_) [10], α-Fe_2_O_3_ [11] and so on. Due to its stable chemical properties [12], environment-friendly features [13], and controllable morphology [14], α-Fe_2_O_3_ can aid in the design of EWAMs. However, Fe_3_O_4_ and γ-Fe_2_O_3_ are easy to reunite, resulting in weak RL performance [15,16]. Any synthesis for massive production should provide a large batch of the primary material of α-Fe_2_O_3_ in this particular case [17]. There are techniques providing large batches, such as the Polyol Method [18]. In the case of α-Fe_2_O_3_, one of the most successful techniques is the laser target evaporation, for which both structural and magnetic properties, including microwave characterization techniques, have been carefully studied [19,20]. α-Fe_2_O_3_ molecules are quite heavy (5.18 g/cm^3^) as traditional EWAMs. In order to solve this problem, a method of loading EWAMs on the surface of low-density materials is proposed. The porous structure of diatoms gives them a light mass (1.9–2.3 g/cm^3^). This porous structure has a huge advantage. It has been reported that core/shell structured nanocomposites based on magnetic nanomaterials exhibit low reflection loss [21,22,23,24,25,26,27]. However, the effective absorption bandwidth of core/shell structure materials is not wide enough [28,29,30]. Therefore, on the basis of the existing research, the light and porous structure of diatomite (De) is used to load a rough sea urchin-like structure of α-Fe_2_O_3_. Hollow micro-spheres and micro-organisms were used as templates to prepare ferrite particles decorated with De through the hydrothermal method [31,32,33,34,35,36,37]. De has the excellent characteristics of biological diatoms and a unique pore structure [38]. Therefore, as a low-density material, De with a hollow double-shell structure is considered to become an excellent material for ferrite particle loading. Lv et al. [39] reported the α-Fe_2_O_3_@CoFe_2_O_4_ core–shell composites. When the thickness of the coating is 2 mm, the RL_min_ of the composite is −60 dB at 16.5 GHz. The frequency bandwidth corresponding to the microwave RL value below −10 dB was up to 5 GHz (13–18 GHz). Guo et al. [40] reported Mg_x_Zn_1−x_ ferrite/diatomite composites. The RL_min_ of the composite is −7.23 dB at 15.4 GHz. In this work, a α-Fe_2_O_3_ nanorod composite was synthesized by the hydrothermal method and further calcination. The results showed that the RL_min_ of α-Fe_2_O_3_-decorated De (α-Fe_2_O_3_@D) could reach −54.24 dB at 11.52 GHz and the matched absorber thickness was 3 mm. The frequency bandwidth corresponding to the microwave RL value below −20 dB was up to 8.24 GHz (9.76–18 GHz).

## 2. Results and Discussion

**Figure 1:**
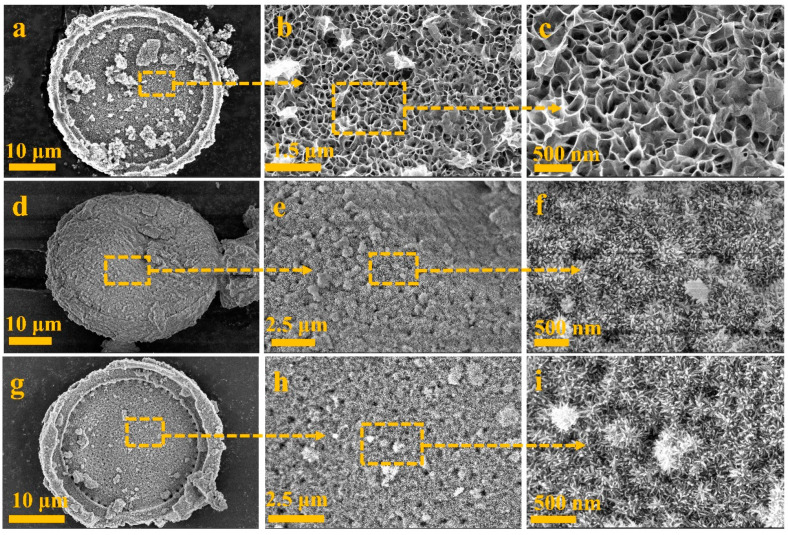
Magnification increases from left to right. SEM images of (**a**–**c**) MnO_2_@D; (**d**–**f**) FeOOH@D; (**g**–**i**) α-Fe_2_O_3_@D.

The surface appearance of prepared MnO_2_@D, FeOOH@D and α-Fe_2_O_3_@D is showed in Figure 1. Energy dispersive spectrometer (EDS) mapping (see Supporting information, Figure S2) of MnO_2_@D determined that the MnO_2_ nanosheets were successfully prepared by the one-step hydrothermal method. The picture in Figure 1a–c indicates the well-distributed growth of MnO_2_ nanosheets on the De. MnO_2_ nanosheets were decorated on De and connected with each other to form a highly uniform surface morphology. SEM images in Figure 1d–f and its EDS mapping (see Supporting information, Figure S3) results further demonstrated that MnO_2_ nanosheets have been completely transferred into sea-urchin-like FeOOHs [41]. The picture in Figure 1g–i indicates that the uniform growth of sea-urchin-like α-Fe_2_O_3_ is similar to FeOOH. EDS (see Supporting information, Figure S4) mapping results further showed that the chemical elements of sea-urchin-like α-Fe_2_O_3_ are mainly made up of O, Fe and K, proving that the sea-urchin-like α-Fe_2_O_3_ specimens were prepared successfully.

**Figure 2:**
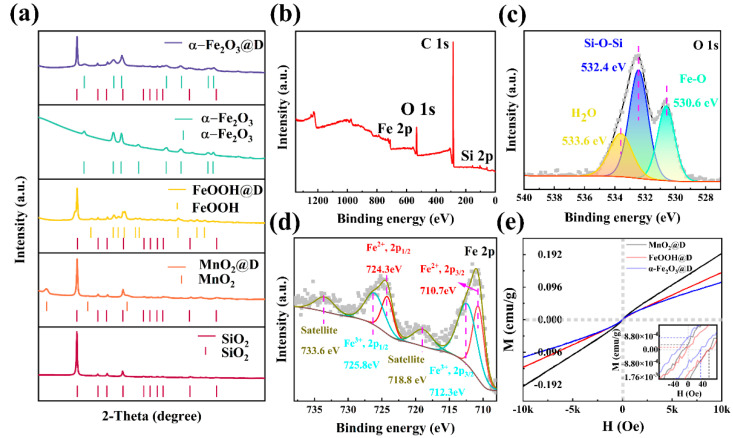
XRD patterns of De, MnO_2_@D, FeOOH@D and α-Fe_2_O_3_@D. (**a**) The XPS of α-Fe_2_O_3_@D: survey; (**b**) O 1s; (**c**) Fe 2p; (**d**) (M−H) loops of MnO_2_@D, FeOOH@D and α-Fe_2_O_3_@D; (**e**) Illustration: the relationship between magnetization and magnetic field of the sample is shown in an enlarged view.

Figure 2a presents the chemical constitution of the De and MnO_2_@D. The four characteristic diffraction peaks at 21.8°, 28.2°, 31°, 36° and 44.4° in De are attributed to the (101), (111), (102), (112) and (202) planes of SiO_2_, respectively, with a quartzite structure (JCPDS card no. 82-0512, a = 4.997 Å, b = 4.973 Å, c = 7.070 Å), which affirms a high crystallization grade. From the XRD patterns of MnO_2_@D, the diffraction peaks at 12.5°, 25.2°, and 37° nearly conform to the normal XRD patterns of birnessite-type manganese oxide crystal (JCPDS card no.80-1098, a = 5.149 Å, b = 2.843 Å, c = 7.176 Å). In particular, the high diffraction peaks of De at 36.1 became wide. The X-ray diffraction patterns in Figure 2a indicate the crystal texture and chemical constitution of FeOOH@D. The obvious broad peaks centered at 33.2°, 34.7°, 36.6°, 40°, 41.2°, 53.2°, 58.9°, and 61.3° correspond to the (130), (021), (111), (121), (140), (221), (151) and (002) of goethite FeOOH, respectively (JCPDS No. 81-0463, a = 4.616 Å, b = 9.955 Å, c = 3.023 Å). In Figure 2a, diffraction peaks of (012), (104), (110), (113), (024), (116), (214), and (300) are similar to the hematite phase (JCPDS card no. 33-0664). This suggests that the FeOOH had been completely transferred into α-Fe_2_O_3_.

To go further to determine the electronic structure, chemical bonding state and composition of α-Fe_2_O_3_@D nanocomposites, XPS analysis was performed. The XPS survey spectrum (Figure 2b) shows that O, Fe and Si elements coexist in α-Fe_2_O_3_@D. The XPS spectra of O1s (Figure 2c) shows three peaks, which were located at 529.7, 531.3 and 532.7 eV. These peaks were assigned as metal–oxygen bonds (Fe–O), silicon–oxygen bonds (Si–O–Si) and surface-adsorbed water (H_2_O), respectively [42,43,44]. There are two main peaks in XPS spectra of the Fe 2p region at 710.7 and 724.3 eV (Figure 2d), which reflect the Fe 2p_3/2_ and Fe 2p_1/2_ orbitals, respectively. Their satellite peaks are clearly distinguishable at 718.8 and 733.6 eV respectively. These peaks belong to Fe^3+^ species [45,46]. The existence of Fe–O further illustrated the successful preparation of α-Fe_2_O_3_. The detailed Rietveld refinement images of samples are shown in the image (See Supporting information, Figure S5). The cell parameters are exhibited in the table (See Supporting information, Table S1).

The magnetic hysteresis loops of the α-Fe_2_O_3_@D compound obtained by a vibrating sample magnetometer are demonstrated in Figure 2e. It displays the applied external field-dependent magnetization hysteresis (M-H) loops of the samples measured between −10 K and 10 K Oe at room temperature. It can be seen that all the samples show similar S-type hysteresis loops, indicating their ferromagnetic behavior. The saturation magnetizations (Ms) of MnO_2_@D, FeOOH@D and α-Fe_2_O_3_@D are 0.19, 0.14, and 0.11 emu·g^−1^, respectively. The composite material is composed of a magnetic shell and a diamagnetic De core. Therefore, the decrease of the Ms value mainly depends on the change of ferrite content on the surface. At the same time, the coercivity (Hc) of samples fluctuated in the range of −7.08~60.75, −1.74~60.39 and −28.41~32.78 Oe. The main reason for the Hc decrease may be that the average particle size increases with the increase of the specific surface area of De during the growth of surface metal oxides.

**Figure 3:**
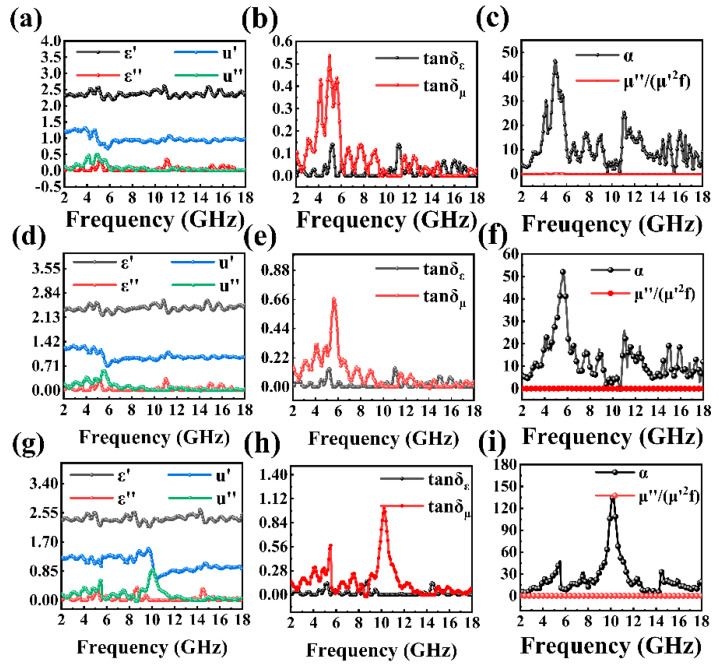
Relevant EM parameters of MnO_2_@D; (**a**–**c**) FeOOH@D; (**d**–**f**) and α-Fe_2_O_3_@D; (**g**–**i**). Frequency dependence of ε′, ε″, μ′ and μ″ (**a**,**d**,**g**); dielectric and loss tangent (**b**,**e**,**h**); attenuation constant α and μ″(μ′)^−2^*f*^−1^ values (**c**,**f**,**i**).

MnO_2_ nanosheets are a dielectric-absorbing material, and their electromagnetic attenuation properties are mainly attributed to their unique lamellae and strip morphologies. However, due to the lamellae structure of pure MnO_2_, itis not suitable for electronic transmission; the real part of the dielectric constant of MnO_2_ (ε′) is smaller than that of FeOOH@D and α-Fe_2_O_3_@D. As antiferromagnetic materials, the magnetic loss of De and MnO_2_ is small enough to be ignored, so the real part (μ′) of the magnetic permeability of MnO_2_@D under alternating electric field is less than one. At the same time the imaginary parts of the dielectric constant (ε″) represent the loss moduli of electric energy and permeability (μ″) represent the loss moduli of magnetic energy. The ε″ and μ″ values of MnO_2_ are low at low frequencies, but show a temporary upward trend at high frequencies, indicating that at low frequencies the electromagnetic wave absorption capacity of MnO_2_ is poor (Figure 3a). The values of μ′ and μ″ can be reflected in the ratio of saturated magnetization of non-magnetic composite particles. In contrast, the specific EM parameters of FeOOH@D are given in Figure 3d. After calcination at 350 °C, the impedance matching of α-Fe_2_O_3_@D was further improved, and the ε″ was increased at a certain frequency (Figure 3j). This change may be caused by various aspects; the porous outer layer of De can be regarded as a micro-ring, while the radio-symmetric sea-urchin-like α-Fe_2_O_3_ can be regarded as numerous antennas that convert electromagnetic waves into vibrating microcurrents. Therefore, a micro current can be generated in the micro circuit, resulting in a dielectric resonance peak in the ε″ curve.

Due to the dielectric loss tangent (ε″/ε′) and magnetic loss tangent (μ″/μ′) value being calculated, the influence of dielectric loss and magnetic loss on the material was reflected (Figure 3b,e,h). As can be seen from the figure (Figure 3b,e,h), from top to bottom, the dielectric loss angle tangent and magnetic loss angle tangent of the three samples all show certain peaks at specific frequencies. The relatively high value of tanδ_ε_ indicates that sea-urchin-like α-Fe_2_O_3_ exhibits a strong dielectric loss, which is caused by dipole polarization, interfacial polarization and related relaxation phenomena. Meanwhile, the formation of new phases due to the synergistic interaction between different compounds is also helpful to improve the electromagnetic wave-absorption performance.

The calculation formula of RL [47,48]:

(1) $$\mathrm{RL}\left(dB\right)=20\log\left|\left(Z_{in}-Z_{0}\right)/\left(Z_{in}+Z_{0}\right)\right|$$

(2) $$Z_{in}=Z_{0}\sqrt{\mu_{r}/\varepsilon_{r}}\tanh\left[j\left(2\pi fd/c\right)\sqrt{\mu_{r}\varepsilon_{r}}\right]$$

**Figure 4:**
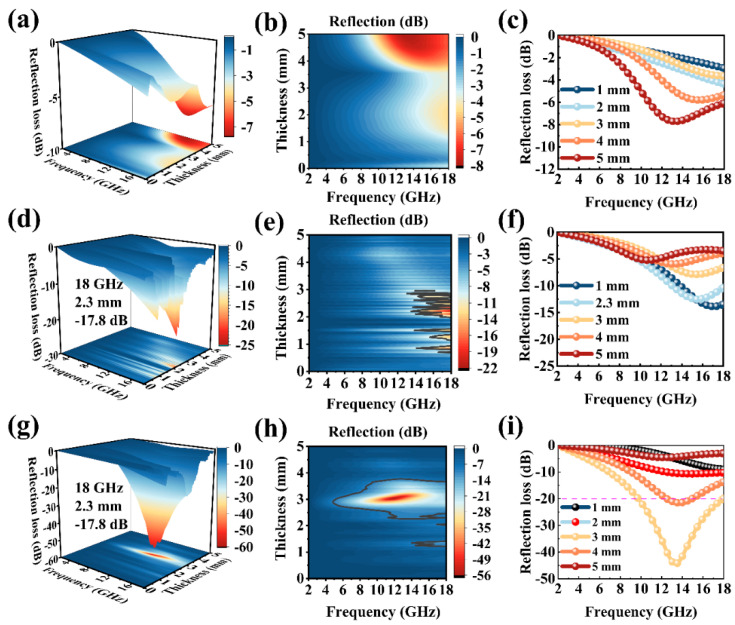
One—dimensional, two—dimensional, and three—dimensional picture of the RL values, which varies with frequency and thickness: (**a**–**c**) MnO_2_@D, (**d**–**f**) FeOOH@D and (**g**–**i**) α-Fe_2_O_3_@D.

The thickness of the specimen is d, the input impedances of the absorbing material and air are *Z_in_* and *Z*_0_. The top to bottom rows represent the RL values of MnO_2_@D to α-Fe_2_O_3_@D, and the three columns on left are 1D plots of the RL values of MnO_2_@D to α-Fe_2_O_3_@D as a function of frequency and thickness; the middle of three columns is 2D and the three columns on right are 3D in Figure 4. MnO_2_ is an impedance-matching material. If the value of MnO_2_@D cannot always be adjusted to −20 dB in the range of 2–18 GHz (Figure 4a–c), it indicates that MnO_2_@D does not have a good absorbing ability and is suitable for adjusting impedance matching materials. FeOOH@D has the potential to become an electromagnetic wave-absorbing material. When the thickness of FeOOH@D is 2.3 mm, the total absorption bandwidth of FeOOH@D is 4.2 GHz at high frequencies (13.8–18 GHz). However, for good absorbent materials, these are not enough. Its mediocre absorbing properties at low frequencies limit the application of FeOOH@D as an absorbing material. After calcination, the magnetic ferrite nanomaterial has a complex geometric structure, which is combined with De to form a double-shell structure. The outer shell is α-Fe_2_O_3_, and the inner shell is De. The enhanced microwave absorption performance is obtained. The electromagnetic absorption performance is gradually enhanced (Figure 4g–i). When the thickness of α-Fe_2_O_3_@D is 3 mm, the f_E_ reaches 8.24 GHz (9.76–18 GHz); meanwhile, the (RL_min_) can reach −54.2 dB. The results show that the thickness of the material, the effective absorption bandwidth and excellent electromagnetic absorption performance allow α-Fe_2_O_3_@D to satisfy the needs of a good-performance absorbing material. Rational material design and morphological construction endow α-Fe_2_O_3_@D with a good adsorption performance. At the same time, the RL_(min)_ and effective absorption bandwidth (RL < −20 dB) of MnO_2_@D, FeOOH@D and α-Fe_2_O_3_@D are shownn in Table 1.

Specifically, good material design should include the following aspects. First, the electric and magnetic energy can be availably removed in the electromagnetic field, because of the synergistic action of the magnetic loss and the dielectric loss. In this work, the α-Fe_2_O_3_ was essential to the magnetic loss component. The influence of the eddy current loss on the absorption results can be roughly analyzed by calculations. The following equation is usually used to judge this effect [41]:

(3) $$\mu^{\prime \prime }=2\pi \mu_{0}{\left(\mu^{\prime }\right)}^{2}\sigma d^{2}f$$

The right-hand side of the equation above is a constant. If the magnetic permeability parameter of the material meets the above formula in the frequency range, the magnetic loss is eddy current loss only. The results show that the value of $\mu^{\prime \prime }{\left(\mu^{\prime }\right)}^{-2}f^{-1}$ fluctuates up and down, not horizontally. Therefore, it can be judged that there are other important reasons such as hysteresis loss, not just eddy current loss. In addition to magnetic loss, dielectric loss is also an important reason for the good absorbing performance of materials. When an electromagnetic wave is incident on a dielectric material, the first microscopic mechanism encountered is the polarization of the medium, which is quite different from that of a conductor with free electrons. In De, the electrons are in bound state, under the action of alternating electric fields. Due to the layered structure of α-Fe_2_O_3_ molecules, the electromagnetic wave produces dielectric polarization phenomena; the center position of the positive and negative charges from superposition into separation generates rotating torque, that is, the electric dipole moment. At the same time, a weak electric field is formed. Therefore, the dielectric loss of the material mainly comes from the rotation and orientation of the dipole during the polarization process, and when the frequency of the applied electric field is consistent with the frequency of the thermal vibration of the molecule, it mainly comes from the resonance.

The loss tangent and attention constant (α) can be calculated directly from the EM parameters. The attention constant can characterize the ability of the absorber to attenuate electromagnetic waves. The higher the value, the stronger the ability of material to dissipate electromagnetic waves. The specific calculation formula is as follows [49]:

(4) $$\alpha =\left(\sqrt{2}\pi f/c\right)\times \sqrt{\left(\mu^{\prime \prime }\varepsilon^{\prime \prime }-\mu^{\prime }\varepsilon^{\prime }\right)+\sqrt{{\left(\mu^{\prime \prime }\varepsilon^{\prime \prime }-\mu^{\prime }\varepsilon^{\prime }\right)}^{2}+{\left(\mu^{\prime }\varepsilon^{\prime \prime }+\mu^{\prime \prime }\varepsilon^{\prime }\right)}^{2}}}$$

The velocity of the electromagnetic wave in vacuum is *c*, The frequency of the incident electromagnetic wave is *f*. As you can see from the Figure 3c,f,i, α-Fe_2_O_3_@D reaches a peak value at about 6 GHz, followed by repeated fluctuations at high frequencies, indicating that there is a good microwave absorption effect at 6 GHz, while there is absorption instability at high frequencies, possibly because the batch stability of De needs to be further improved.

**Figure 5:**
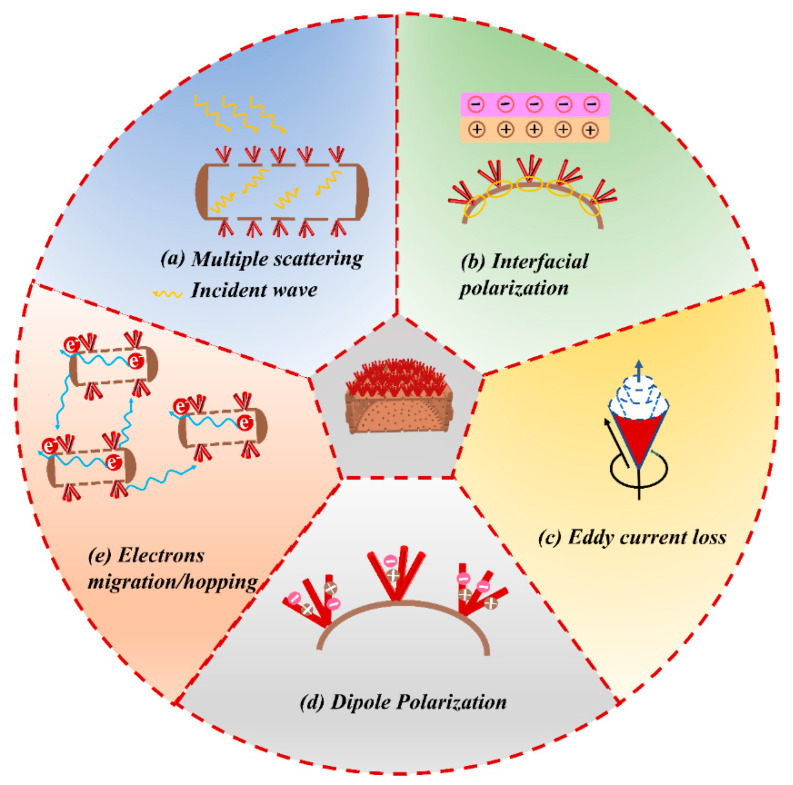
Microwave absorption mechanism of α-Fe_2_O_3_@D composite materials.

The microwave absorption mechanism of α-Fe_2_O_3_@D composites is shown in Figure 5. At low frequencies, ferrites including α-Fe_2_O_3_ exhibit domain wall resonance with increasing magnetic loss. The defects of α-Fe_2_O_3_ can induce local dipole polarization. Electron migrate in α-Fe_2_O_3_. At the same time, the constancy in the values of ε′ indicates the existence of a polarization process, that is, the oscillations of the electric dipole moment coincide or are slightly out of phase with the microwave frequency. The most possible mechanism in this frequency range is orientational polarization [50,51]. First, there are abundant α-Fe_2_O_3_-De interfaces in the α-Fe_2_O_3_@D core–shell structure that promote the aggregation and transfer of free electrons to the interface. This accumulation leads to interfacial polarization relaxation, which is favorable for microwave dissipation into other energy forms. Second, due to the closed current lines formed on the surface of α-Fe_2_O_3_, the induced current in the α-Fe_2_O_3_@D core–shell composite material leads to energy loss, and eddy current loss occurs. The interaction between α-Fe_2_O_3_ and De contributes to the formation of natural resonance and exchange resonance, which is also one of the reasons for the occurrence of magnetic loss. Finally, the abundant sea-urchin-like α-Fe_2_O_3_ on the surface of De can scatter electromagnetic waves and deflect the direction of the incident electromagnetic waves in the same direction, resulting in electromagnetic wave energy offset.

**Figure 6:**
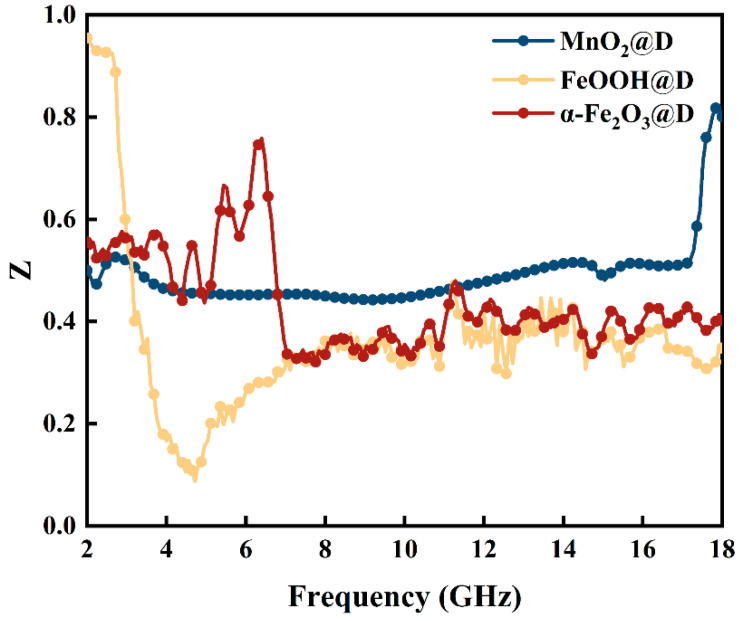
Frequency dependence of impedance matching *Zr* of the MnO_2_@D, FeOOH@D and α-Fe_2_O_3_@D composite material.

In Figure 6, the values of $Z_{r}=\sqrt{\mu_{r}/\varepsilon_{r}}$ increase and then decrease as frequency increases. This may be because α-Fe_2_O_3_@D creates the right eddies when electromagnetic waves reach the surface. This matches the multiple reflection of De and enhances the incidence and absorption of electromagnetic waves. Due to the skin effect of dense α-Fe_2_O_3_@D, the impedance-matching performance decreases, and the overflow eddy current breaks the equilibrium.

Compared with other De-based EWAM, the fabricated α-Fe_2_O_3_@D in this work contained two major advantages. Firstly, De has the characteristics of size control; De has an excellent size, otherwise it is not conducive to the adhesion of other load materials, and if De is not uniform, it will affect the uniformity of composite materials. Additionally, the double-shell structure is connected by tight chemical bonds, which ensures the safety of the double-shell structure. Therefore, five samples with De-based were selected to compare their absorbing properties with our samples, as shown in Table 2. The consequences indicated that the RL_min_ of α-Fe_2_O_3_@D is −54.2 dB. The wide effective absorption bandwidth (8.24 GHz) at a thinner thickness (3 mm) demonstrated that the EWAM has the characteristics of being lightweight and of high efficiency, so its application prospect is very broad in the future.

## 3. Methods and Materials

α-Fe_2_O_3_@D, the De, potassium hypermanganate [KMnO_4_], ferrous sulfate [FeSO_4_·7H_2_O], and ethylene glycol [HOCH_2_—CH_2_OH] were purchased from Aladdin. In this work, all the lab supplies used were analytically pure and used without further purification.

### 3.1. Synthesis of MnO_2_ Nanosheets on De

The one-step hydrothermal method was used to prepare MnO_2_-decorated De (MnO_2_@D). Typically, 100 mg of De and KMnO_4_ homogeneous solution (70 mL, 0.05 M) was put into a 100 mL autoclave. The Teflon-lined stainless-steel autoclave was subsequently sealed and maintained in a homogeneous reactor at 160 °C for 24 h. The solid precipitates were washed with distilled water and alcohol, and dried in atmosphere at 60 °C for 12 h.

### 3.2. Preparation of Sea-Urchin-Like FeOOH on De

The sea-urchin-like FeOOH on De was achieved via a hydrothermal method. An amount of 110 mg FeSO_4_·7H_2_O was dissolved into 70 mL of solvent including distilled water and ethylene glycol (V_distilled water_/V_ethylene glycol_ = 7/1), then the mixed solvent was stirred for about 10 min. Next, 80 mg MnO_2_@D and 70 mL FeSO_4_·7H_2_O mixed solution were transferred into a 100 mL Teflon-lined stainless-steel autoclave and maintained at 120 °C for 2 h in a homogeneous reactor. The autoclave was cooled to 25 °C, after the hydrothermal reaction finished. The samples were washed with distilled water and alcohol; after that, the washed samples were dried in atmosphere at 60 °C for 12 h.

### 3.3. Preparation of α-Fe_2_O_3_@D

α-Fe_2_O_3_@D was obtained by calcination of FeOOH@D at 350 °C for 2 h under O_2_ atmosphere. For the preparation flow chart of α-Fe_2_O_3_@D, (see Supporting information, Figure S1)

### 3.4. Characterization of Material

The nanostructures and surface appearance of the material were characterized by scanning electron microscopy (FIB/SEM, ZEISS AURIGA). The crystal texture and chemical constitution of the nanostructures were confirmed by X-ray diffraction (XRD, PANalytical X’Pert Powder), and the data of XRD results were analyzed with the JADE 6.0 software. The data of X-ray photoelectron spectroscopy (XPS) spectra results were obtained on a spectrometer (ESCALAB 250Xi). To measure the permittivity and permeability of α-Fe_2_O_3_@D, the compounds were prepared by mixing the as-prepared α-Fe_2_O_3_@D with melted paraffin according to the mass fractions of 20%, then each mixture was pressed to form a cylindrical toroidal with an outer diameter of 7.0 mm, inner diameter of 3.04 mm and thickness of 3.0 mm. The relative complex permeability ($\varepsilon_{r}=\varepsilon^{\prime }-j\varepsilon^{\prime \prime }$) and permittivity ($\mu_{r}=\mu^{\prime }-j\mu^{\prime \prime }$) were resulted by the coaxial line method with an Agilent 85071E vector network analyzer in the frequency range of 2.0–18.0 GHz.

## 4. Conclusions

The conclusion of the paper is updated as follows: in summary, a series of three samples with a double hull configuration were systematically designed by the hydrothermal method. By decorating De with α-Fe_2_O_3_ nanowires, the electrical conductivity, dielectric properties and impedance matching of the De were improved effectively. Therefore, α-Fe_2_O_3_@D exhibits enhanced EM wave-absorption performance thanks to its optimal dielectric performance and impedance matching. The RL_min_ of α-Fe_2_O_3_@D could reach −54.2 dB at 11.52 GHz and the matched absorber thickness was 3 mm. The frequency bandwidth corresponding to the microwave RL value below −20 dB was up to 8.24 GHz (9.76–18 GHz). α-Fe_2_O_3_@D synthesized in this paper has been applied to the electromagnetic wave absorption field for the first time, providing an important reference for eliminating the influence of electromagnetic interference on the human body and equipment.

## Figures and Tables

**Table 1:** RL(min) and effective absorption bandwidths (RL < −20 dB) of MnO2@D, FeOOH@D and α-Fe_2_O_3_@D.

Sample Name	RL_(min)_	Effective Absorption Bandwidth (RL < −20 dB)
MnO_2_@D	−7.9 dB	0
FeOOH@D	−17.8 dB	0
α-Fe_2_O_3_@D	−54.2 dB	8.24 GHz

**Table 2:** Comparison with other De basic materials.

Sample Name	Percentage (wt.%)	RL_min_ (dB)	Absorberthickness (mm)	EAB(RL < −10 dB) (GHz)	Reference
Fe_2_O_4_/α-Fe_2_O_3_	20	−52.69	3	5.36	[52]
α-Fe_2_O_3_-graphene	15	−30.6	4	5.5	[53]
α-Fe_2_O_3_/OPEFB fiber/PCL	25	−38	6	3	[54]
RGO/PANI/α-Fe_2_O_3_@SiO_2_ with 1:4	16.7	−31.06	5	2	[55]
RGO/PANI/α-Fe_2_O_3_@SiO_2_ with 1:6	16.7	−25.88	4	3	[55]
α-Fe_2_O_3_@D	20	−54.2	3	8.24	this work

## Data Availability

Data of the compounds are available from the authors.

## Supplementary Materials

The following supporting information can be downloaded at: https://www.mdpi.com/article/10.3390/ijms23169362/s1.

## References

[1] Girgert R., Grundker C., Emons G., Hanf V. (2008). Electromagnetic fields alter the expression of estrogen receptor cofactors in breast cancer cells. Bioelectromagnetics.

[2] Liu X.G., Jiang J.J., Geng D.Y., Li B.Q., Han Z., Liu W., Zhang Z.D. (2009). Dual nonlinear dielectric resonance and strong natural resonance in Ni/ZnO nanocapsules. Appl. Phys. Lett..

[3] Wang Y., Li T., Zhao L., Hu Z., Gu Y. (2011). Research progress on nanostructured radar absorbing materials. Energy Sci. Eng..

[4] Qin F.X., Peng H.X. (2013). Ferromagnetic microwires enabled multifunctional composite materials. Prog. Mater. Sci..

[5] Yang P.-A., Huang Y., Li R., Huang X., Ruan H., Shou M., Li W., Zhang Y., Li N., Dong L. (2022). Optimization of Fe@Ag core–shell nanowires with improved impedance matching and microwave absorption properties. Chem. Eng. J..

[6] Wang D.-S., Mukhtar A., Wu K.-M., Gu L., Cao X. (2019). Multi-segmented nanowires: A high tech bright future. Materials.

[7] Almessiere M.A., Guner S., Slimani Y., Hassan M., Baykal A., Gondal M.A., Baig U., Trukhanov S.V., Trukhanov A.V. (2021). Structural and Magnetic Properties of Co_0.5_Ni_0.5_Ga_0.01_Gd_0.01_Fe_1.98_O_4_/ZnFe_2_O_4_ Spinel Ferrite Nanocomposites: Comparative Study between Sol-Gel and Pulsed Laser Ablation in Liquid Approaches. Nanomaterials.

[8] Yakovenko O.S., Matzui L.Y., Vovchenko L.L., Oliynyk V.V., Zagorodnii V.V., Trukhanov S.V., Trukhanov A.V. (2021). Electromagnetic Properties of Carbon Nanotube/BaFe_12-x_Ga_x_O_19_/Epoxy Composites with Random and Oriented Filler Distributions. Nanomaterials.

[9] Trukhanov A., Turchenko V., Bobrikov I., Trukhanov S., Kazakevich I., Balagurov A. (2015). Crystal structure and magnetic properties of the BaFe_12−x_Al_x_O_19_ (x = 0.1–1.2) solid solutions. J. Magn. Magn. Mater..

[10] Karpinsky D.V., Silibin M.V., Trukhanov S.V., Trukhanov A.V., Zhaludkevich A.L., Latushka S.I., Zhaludkevich D.V., Khomchenko V.A., Alikin D.O., Abramov A.S. (2020). Peculiarities of the crystal structure evolution of BiFeO_3_–BaTiO_3_ ceramics across structural phase transitions. Electromagn. Prop..

[11] Vinnik D., Zhivulin V., Sherstyuk D., Starikov A.Y., Zezyulina P., Gudkova S., Zherebtsov D., Rozanov K., Trukhanov S., Astapovich K. (2021). Electromagnetic properties of zinc–nickel ferrites in the frequency range of 0.05–10 GHz. Mater. Today Chem..

[12] Lee E.-T., Jang G.-E., Kim C.K., Yoon D.-H. (2001). Fabrication and gas sensing properties of α-Fe_2_O_3_ thin film prepared by plasma enhanced chemical vapor deposition (PECVD). Sens. Actuators B Chem..

[13] Hu J., Chen G.H., Lo I.M.C. (2006). Selective removal of heavy metals from industrial wastewater using maghemite nanoparticle: Performance and mechanisms. J. Environ. Eng..

[14] Liao L., Zheng Z., Yan B., Zhang J., Gong H., Li J., Liu C., Shen Z., Yu T. (2008). Morphology controllable synthesis of α-Fe_2_O_3_ 1D nanostructures: Growth mechanism and nanodevice based on single nanowire. J. Phys. Chem. C.

[15] Javid M., Zhou Y.L., Wang D.X., Li D., Shi G.M., Kim U., Zhou L., Dong X.L., Zhang Z.D. (2018). Magnetic Behavior, Electromagnetic Multiresonances, and Microwave Absorption of the Interfacial Engineered Fe@FeSi/SiO_2_ Nanocomposite. ACS Appl. Nano Mater..

[16] Liu P., Gao S., Wang Y., Huang Y., Wang Y., Luo J. (2019). Core–shell CoNi@graphitic carbon decorated on B, N-codoped hollow carbon polyhedrons toward lightweight and high-efficiency microwave attenuation. ACS Appl. Mater. Interfaces.

[17] Grossman J.H., McNeil S.E. (2012). Nanotechnology in cancer medicine. Phys. Today.

[18] Ansari S.A.M.K., Ficiarà E., Ruffinatti F.A., Stura I., Argenziano M., Abollino O., Cavalli R., Guiot C., D’Agata F. (2019). Magnetic iron oxide nanoparticles: Synthesis, characterization and functionalization for biomedical applications in the central nervous system. Materials.

[19] Osipov V., Platonov V., Uimin M., Podkin A. (2012). Laser synthesis of magnetic iron oxide nanopowders. Tech. Phys..

[20] Safronov A., Beketov I., Komogortsev S., Kurlyandskaya G., Medvedev A., Leiman D., Larrañaga A., Bhagat S. (2013). Spherical magnetic nanoparticles fabricated by laser target evaporation. AIP Adv..

[21] Che R.C., Peng L.M., Duan X.F., Chen Q., Liang X.L. (2004). Microwave absorption enhancement and complex permittivity and permeability of Fe encapsulated within carbon nanotubes. Adv. Mater..

[22] Chen Y.J., Xiao G., Wang T.S., Ouyang Q.Y., Qi L.H., Ma Y., Gao P., Zhu C.L., Cao M.S., Jin H.B. (2011). Porous Fe_3_O_4_/Carbon Core/Shell Nanorods: Synthesis and Electromagnetic Properties. J. Phys. Chem. C.

[23] Cao J., Fu W., Yang H., Yu Q., Zhang Y., Liu S., Sun P., Zhou X., Leng Y., Wang S. (2009). Large-scale synthesis and microwave absorption enhancement of actinomorphic tubular ZnO/CoFe_2_O_4_ nanocomposites. J. Phys. Chem. B.

[24] An Z., Pan S., Zhang J. (2009). Facile Preparation and Electromagnetic Properties of Core− Shell Composite Spheres Composed of Aloe-like Nickel Flowers Assembled on Hollow Glass Spheres. J. Phys. Chem. C.

[25] Ohlan A., Singh K., Chandra A., Dhawan S.K. (2010). Microwave absorption behavior of core-shell structured poly (3,4-ethylenedioxy thiophene)-barium ferrite nanocomposites. ACS Appl. Mater. Interfaces.

[26] Chen Y.J., Zhang F., Zhao G.G., Fang X.Y., Jin H.B., Gao P., Zhu C.L., Cao M.S., Xiao G. (2010). Synthesis, Multi-Nonlinear Dielectric Resonance, and Excellent Electromagnetic Absorption Characteristics of Fe_3_O_4_/ZnO Core/Shell Nanorods. J. Phys. Chem. C.

[27] Wang D., Mukhtar A., Humayun M., Wu K., Du Z., Wang S., Zhang Y. (2022). A Critical Review on Nanowire-Motors: Design, Mechanism and Applications. Chem. Rec..

[28] Yun X.J., Wu Q.F., Feng L., Shen J.C., Chen J., Chu P.K., Liu L.Z., Wu X.L. (2020). Microwave absorption enhancement of e-Fe_3_O_4_@C microspheres by core surface modification. J. Alloys Compd..

[29] Zhu Q., Zhang X., Wang X., Wu X., Zhang Z., Shen J. (2022). Hydrothermal self-assembled Fe_3_O_4_/CA core-shell composites for broadband microwave absorption. J. Magn. Magn. Mater..

[30] Liu S.D., Meng X.W., Wang Z.Z., Li Z.H., Yang K. (2019). Enhancing microwave absorption by constructing core/shell TiN@TiO_2_ heterostructures through post-oxidation annealing. Mater. Lett..

[31] Fu W.Y., Liu S.K., Fan W.H., Yang H.B., Pang X.F., Xu J., Zou G.T. (2007). Hollow glass microspheres coated with CoFe_2_O_4_ and its microwave absorption property. J. Magn. Magn. Mater..

[32] Mu G.H., Pan X.F., Shen H.G., Gu M.Y. (2007). Preparation and magnetic properties of composite powders of hollow microspheres coated with barium ferrite. Mater. Sci. Eng. A.

[33] Li K., Liu X., Zheng T., Jiang D., Zhou Z., Liu C., Zhang X., Zhang Y., Losic D. (2019). Tuning MnO_2_ to FeOOH replicas with bio-template 3D morphology as electrodes for high performance asymmetric supercapacitors. Chem. Eng. J..

[34] Li K., Feng S., Jing C., Chen Y., Liu X., Zhang Y., Zhou L. (2019). Assembling a double shell on a diatomite skeleton ternary complex with conductive polypyrrole for the enhancement of supercapacitors. ChemComm.

[35] Wang T., Li K., Le Q., Zhu S., Guo X., Jiang D., Zhang Y. (2021). Tuning parallel manganese dioxide to hollow parallel hydroxyl oxidize iron replicas for high-performance asymmetric supercapacitors. J. Colloid Interface Sci..

[36] Li K., Hu Z., Zhao R., Zhou J., Jing C., Sun Q., Rao J., Yao K., Dong B., Liu X. (2021). A multidimensional rational design of nickel–iron sulfide and carbon nanotubes on diatomite via synergistic modulation strategy for supercapacitors. J. Colloid Interface Sci..

[37] Li K., Teng H., Dai X., Wang Y., Wang D., Zhang X., Yao Y., Liu X., Feng L., Rao J. (2022). Atomic scale modulation strategies and crystal phase transition of flower-like CoAl layered double hydroxides for supercapacitors. CrystEngComm.

[38] Zhang Y., Cai R., Wang D., Li K., Sun Q., Xiao Y., Teng H., Huang X., Sun T., Liu Z. (2022). Lightweight, Low-Cost Co_2_SiO_4_@diatomite Core-Shell Composite Material for High-Efficiency Microwave Absorption. Molecules.

[39] Lv H., Liang X., Cheng Y., Zhang H., Tang D., Zhang B., Ji G., Du Y. (2015). Coin-like α-Fe_2_O_3_@ CoFe_2_O_4_ core–shell composites with excellent electromagnetic absorption performance. ACS Appl. Mater. Interfaces.

[40] Guo W., Wang S., Ren Q., Jin Z., Ding Y., Xiong C., Li J., Chen J., Zhu Y., Oh W.-C. (2022). Microwave absorption and photocatalytic activity of Mg_x_Zn_1−x_ ferrite/diatomite composites. J. Korean Ceram. Soc..

[41] Wu Z.C., Pei K., Xing L.S., Yu X.F., You W.B., Che R.C. (2019). Enhanced Microwave Absorption Performance from Magnetic Coupling of Magnetic Nanoparticles Suspended within Hierarchically Tubular Composite. Adv. Funct. Mater..

[42] Kuang P.Y., Zhang L.Y., Cheng B., Yu J.G. (2017). Enhanced charge transfer kinetics of Fe_2_O_3_/CdS composite nanorod arrays using cobalt-phosphate as cocatalyst. Appl. Catal. B.

[43] Rui Q., Wang L., Zhang Y., Feng C., Zhang B., Fu S., Guo H., Hu H., Bi Y. (2018). Synergistic effects of P-doping and a MnO_2_ cocatalyst on Fe_2_O_3_ nanorod photoanodes for efficient solar water splitting. J. Mater. Chem. A.

[44] Bu X.B., Gao Y.X., Zhang S.H., Tian Y. (2019). Amorphous cerium phosphate on P-doped Fe_2_O_3_ nanosheets for efficient photoelectrochemical water oxidation. Chem. Eng. J..

[45] Zheng P.L., Zhang Y., Dai Z.F., Zheng Y., Dinh K.N., Yang J., Dangol R., Liu X.B., Yan Q.Y. (2018). Constructing Multifunctional Heterostructure of Fe_2_O_3_@Ni_3_Se_4_ Nanotubes. Small.

[46] Bayazit M.K., Cao E., Gavriilidis A., Tang J. (2016). A microwave promoted continuous flow approach to self-assembled hierarchical hematite superstructures. Green Chem..

[47] Zhou W.M., Zhang J., Liu Y.B., Li X.L., Niu X.M., Song Z.T., Min G.Q., Wan Y.Z., Shi L.Y., Feng S.L. (2008). Characterization of anti-adhesive self-assembled monolayer for nanoimprint lithography. Appl. Surf. Sci..

[48] Huang B., Yue J.L., Wei Y.S., Huang X.Z., Tang X.Z., Du Z.J. (2019). Enhanced microwave absorption properties of carbon nanofibers functionalized by FeCo coatings. Appl. Surf. Sci..

[49] Cao M.S., Wang X.X., Cao W.Q., Fang X.Y., Wen B., Yuan J. (2018). Thermally Driven Transport and Relaxation Switching Self-Powered Electromagnetic Energy Conversion. Small.

[50] Ahmad S.H., Abdullah M.H., Hui D., Yusoff A.N., Puryanti D. (2010). Magnetic and microwave absorbing properties of magnetite–thermoplastic natural rubber nanocomposites. J. Magn. Magn. Mater..

[51] Kurlyandskaya G., Safronov A., Bhagat S., Lofland S., Beketov I., Prieto L.M. (2015). Tailoring functional properties of Ni nanoparticles-acrylic copolymer composites with different concentrations of magnetic filler. J. Appl. Phys..

[52] Vickers N.J. (2017). Animal Communication: When I’m Calling You, Will You Answer Too?. Curr. Biol..

[53] Wang L., Bai X., Wang M. (2018). Facile preparation, characterization and highly effective microwave absorption performance of porous α-Fe_2_O_3_ nanorod–graphene composites. J. Mater. Sci. Mater. Electron..

[54] Mensah E.E., Abbas Z. (2022). Experimental and Computational Study of the Microwave Absorption Properties of Recycled α-Fe_2_O_3_/OPEFB Fiber/PCL Multi-Layered Composites. J. Mater. Sci. Chem. Eng..

[55] Zhang N., Huang Y., Wang M. (2018). Synthesis of graphene/thorns-like polyaniline/alpha-Fe_2_O_3_@SiO_2_ nanocomposites for lightweight and highly efficient electromagnetic wave absorber. J. Colloid Interface Sci..

