# Supplementary material for: Microwave Absorption of α-Fe2O3@diatomite Composites
Source: Int J Mol Sci. 2022 Aug 19;23(16):9362. doi: 10.3390/ijms23169362 (PMC9409334; doi:10.3390/ijms23169362)
Supplement: Supplementary file 1 [file ijms-23-09362-s001.zip › ijms-1821955-supplementary.pdf]

Microwave Absorption of α–Fe_2O_3@diatomite Composites

Chenzhi Zhang [a], Dashuang Wang [a], Lichao Dong [b],Kailin Li [a], Yifan Zhang [a], Pingan Yang [c], Shuang Yi [a], Xingjian Dai [a], Changqing Yin [a]， Zhilan Du [a], Xinfang Zhang [a], Quan Zhou [a], Zhiyu Yi [a],Jinsong Rao [a*], Yuxin Zhang [a*]

a College of Material Science and Engineering, Chongqing University, Chongqing 400044, China;
b Aerospace Institute of Advanced Materials & Processing Technology, Beijing 100074, China;
c School of Automation, Chongqing University of Posts and Telecommunications, Chongqing 400065, China;

*Corresponding author.

E-mail: rjs@cqu.edu.cn; zhangyuxin@cqu.edu.cn

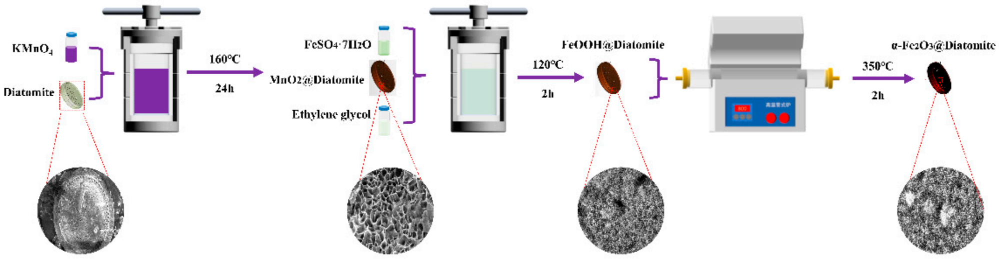

Figure. S1.　Preparation flow chart of α-Fe_2O_3@D

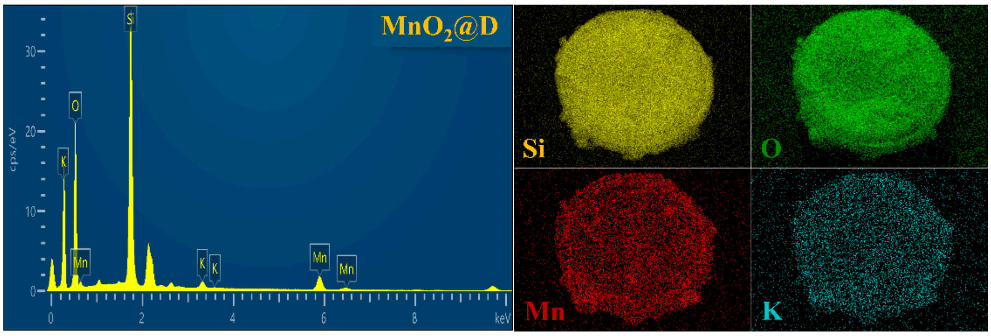

Figure. S2. The EDS images of MnO_2@D

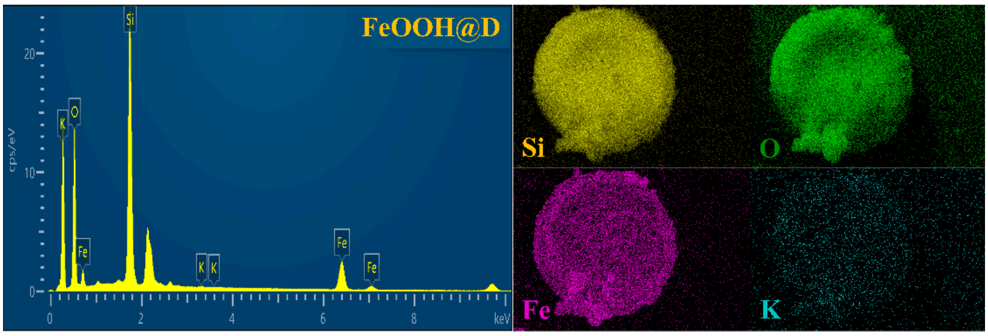

Figure. S3. The EDS images of FeOOH@D

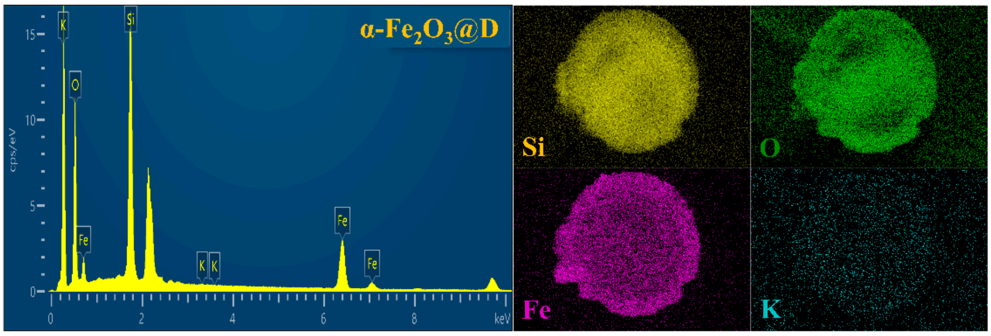

Figure. S4. The EDS images of α-Fe_2O_3@D

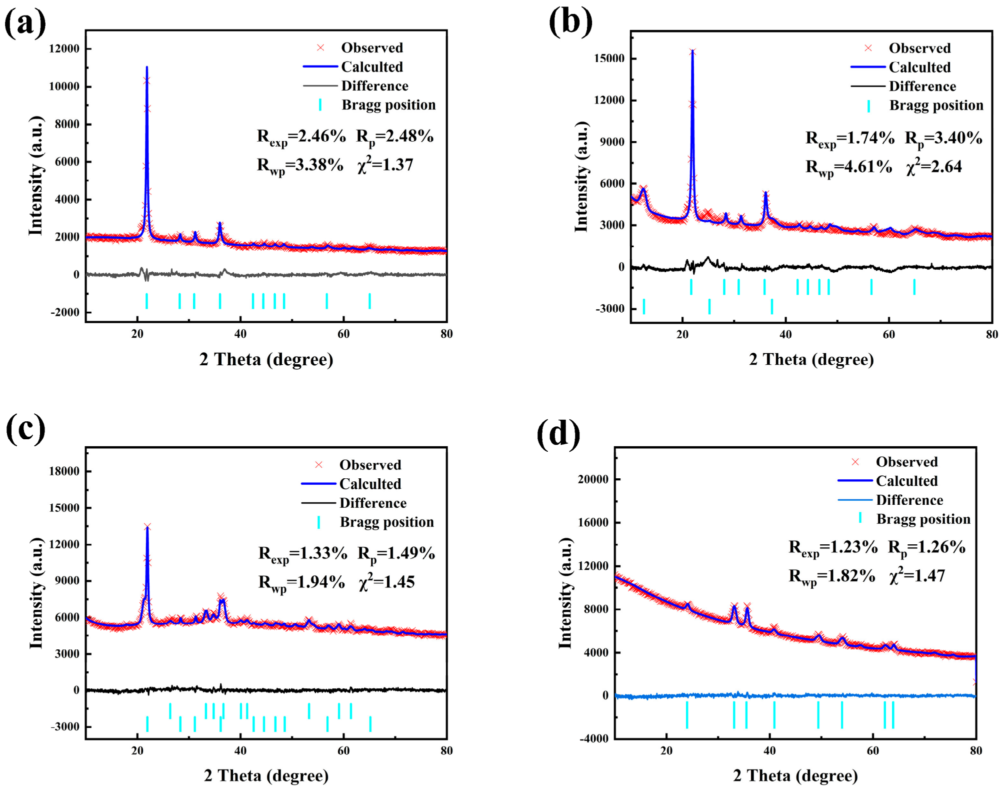

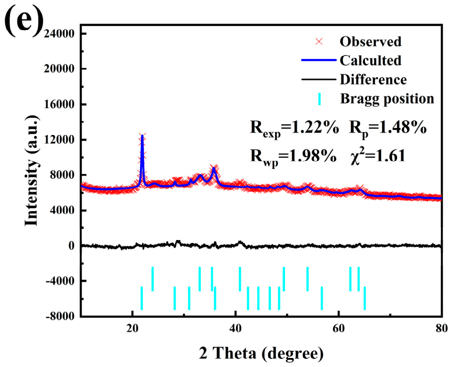

Figure. S5. The detailed Rietveld refinement images of De(a), MnO_2@D(b), FeOOH@D(c), α-Fe_2O_3(d), α-Fe_2O_3@D(e).

Table. S1. Cell parameters are shown in the following table.

Sample	a [Å]	b [Å]	c [Å]	α [°]	β [°]	γ [°]	Volume [Å^3]	R_{exp}	R_p	R_{wp}	χ^2
SiO_2	4.98	4.98	6.99	90	90	90	173.99	2.46%	2.48%	3.38%	1.37
MnO_2@D	9.81	9.81	2.84	90	90	90	274.26	1.74%	3.40%	4.61%	2.64
FeOOH@D	9.95	3.02	4.61	90	90	90	139.04	1.33%	1.49%	1.94%	1.45
Fe_2O_3	8.72	5.03	5.39	90	121.7	90	201.38	1.23%	1.26%	1.82%	1.47
Fe_2O_3@D	5.03	5.03	13.83	90	90	120	303.65	1.22%	1.48%	1.98%	1.61
